# Molecular Mechanisms of the Therapeutic Effect of Selenium Nanoparticles in Hepatocellular Carcinoma

**DOI:** 10.3390/cells13131102

**Published:** 2024-06-26

**Authors:** Elena G. Varlamova

**Affiliations:** Institute of Cell Biophysics of the Russian Academy of Sciences, Federal Research Center “Pushchino Scientific Center for Biological Research of the Russian Academy of Sciences”, 142290 Pushchino, Russia; 1928lv@mail.ru

**Keywords:** hepatocellular carcinoma, selenium nanoparticles, apoptosis, autophagy

## Abstract

This review describes and summarizes, for the first time, the molecular mechanisms of the cytotoxic effect of selenium nanoparticles of various origins on hepatocellular carcinoma cells. The text provides information from recent years indicating the regulation of various signaling pathways and endoplasmic reticulum stress by selenium nanoparticles; the pathways of cell death of liver cancer cells as a result of exposure to selenium nanoparticles are considered. Particular attention is paid to the participation of selenoproteins and selenium-containing thioredoxin reductases and glutathione peroxidases in these processes. Previously, there were no reviews that fully reflected the cytotoxic effects of selenium nanoparticles specifically in hepatocellular carcinoma, despite the fact that many reviews and experimental articles have been devoted to the causes of this disease and the molecular mechanisms of regulation of cytotoxic effects by other agents. The relevance of this review is primarily explained by the fact that despite the development of various drugs and approaches for the treatment and prevention of hepatocellular carcinoma, this disease is still the fourth leading cause of death in the world. For this reason, a complete understanding of the latest trends in the treatment of oncology of various etiologies, especially hepatocellular carcinoma, is extremely important.

## 1. Introduction

Hepatocellular carcinoma (HCC) is the most common primary liver cancer and the fourth leading cause of death worldwide, the main cause of which is cirrhosis, regardless of its etiology; other common causes include carcinogens and genetic disorders [1,2]. Cancer development is very often characterized by the coevolution of cancer and immune cells [3,4]. HCC is not just a mixture of cells and extracellular matrix but several cell types that interact with each other and with surrounding tissues, creating a complex network of interactions. Multikinase inhibitors such as sorafenib, lenvatinib, regorafenib and cabozantinib, as well as the vascular endothelial growth factor (VEGF) inhibitor ramucirumab, are widely used clinically for the treatment of HCC [5,6,7,8]. In recent years, new therapeutic strategies have also been developed, such as immunosuppressive cancer therapy based on immune checkpoint inhibitors, with the combination of this method with the drug ramucirumab in advanced HCC showing better results compared with the widely used multikinase inhibitor sorafenib [9], while the combination of atezolizumab and bevacizumab is now positioned as first-line therapy for patients with advanced HCC. Increasingly, nanotechnology has recently been used to treat oncology of various etiologies; in particular, a lot of work has been devoted to the study of nanoparticles and nanocomplexes for the treatment of HCC, since they provide targeted delivery of drugs, increasing their therapeutic effectiveness. Among nanoparticles based on metals and nonmetals in HCC, the most widely used are gold nanoparticles, which have a high ability to bind to proteins and lipids on the surface of cancer cells [10]; iron nanoparticles, which are used in magnetic resonance analysis of HCC due to their interaction with external magnetic fields; and silver nanoparticles doped with curcumin, which have exhibited powerful antioxidant, anticarcinogenic, anti-inflammatory and antiangiogenic activity [11].

To date, sufficient research has been accumulated on the role of selenium nanoparticles (SeNPs) in the treatment of liver cancer. Such research is usually divided into two areas, namely the study of chemoprotective and chemosensitizing activities and the study of direct cytotoxic activity against liver cancer cells [12,13,14]. Almost always, treatment with SeNPs is accompanied by normalization of reactive oxygen species (ROS) and lipid peroxidation (LPO) levels; a decrease in the blood levels of alanine aminotransferase (ALT) and aspartate aminotransferase (AST) enzymes; and an increase in the activity of enzymes such as superoxide dismutase (SOD), catalase (CAT), glutathione (GSH), glutathione peroxidase (GPX) and glutathione S-transferase (GST) [15,16,17].

This review is devoted to describing the latest data regarding the molecular mechanisms of the cytotoxic effect of selenium-based nanoparticles towards HCC. For the first time, generalized information on the regulation of endoplasmic reticulum stress (ER-stress) and ER-resident selenoproteins activated in HCC by SeNPs is provided, and the putative functions of these selenoproteins in the progression or inhibition of HCC are described. Despite the fact that, recently, more and more information has appeared on the role of SeNPs in the treatment of HCC, no review has considered the molecular mechanisms of the regulation of hepatocarcinogenesis controlled by these nanoparticles and combined all the available information on their cytotoxicity in relation to HCC, which was the global goal of this review.

## 2. The “Therapeutic Window” of SeNPs Determines Their Hepatoprotective Functions

It is known that the antioxidant activity is one of the main functions of Se-containing compounds of organic and inorganic nature, including selenoproteins and Se-containing nanoparticles. Antioxidants can prevent the development of cancer by influencing the cell cycle, inflammation, proliferation, tumor invasiveness, apoptosis mechanisms, etc. [18]. On the other hand, ROS are essential for the adaptive response of cells, as well as their survival. In many ways, the answer to the question about the dangers and benefits of antioxidants lies in the used concentration range, depending on which they can behave as either pro- or antioxidants. It is known that Se can cause various toxic symptoms and has a narrow safe dosage range. In infectious, oncological and toxic diseases, the daily requirement for Se to protect the body increases from 50–100 μg to 600 μg/day per person, while the toxic dose is 900 μg/day per person [19].

Among all Se-containing agents, SeNPs have the least risk of toxicity, but the mechanisms they activate may differ depending on the cell type (normal or cancerous), the type of cancer cells, the dosage and the physicochemical properties of the nanoparticles themselves [20,21,22,23,24,25]. A number of studies have demonstrated that SeNPs can induce apoptotic death of cancer cells, for example, through the p53 and AKT pathways [26,27], by activating the mitochondria-mediated pathway [28,29], by inhibiting the EGFR (epidermal growth factor receptor)-mediated PI3K/AKT pathways and Ras/Raf/MEK/ERK and through activation of MAPK and the caspase-3 signaling pathway [30,31]. All these works highlight the anticancer potential of nanoparticles.

An alternative mechanism for the therapeutic properties of SeNPs is autophagic death of cancer cells, which can be triggered by enhancing signaling pathways associated with beclin-1 [32], suppressing the p62 protein [33,34,35] and by inhibiting the PI3K/AKT/mTOR pathway. On the other hand, there are studies indicating inhibition of autophagy by disrupting the function of lysosomes, alkalizing the lysosomal environment and blocking the late stage of autophagy [36,37].

However, it should be emphasized that excessive doses of SeNPs certainly have a cytotoxic effect [38,39]. Thus, in a carbon tetrachloride-induced inflammatory model in rats, high doses of SeNPs (10 mg/100 g) stabilized by a natural sulfated polysaccharide caused serious disorders associated with dysfunction of the system for excreting excess Se in the form of its methylated derivatives by the kidneys. This led to excess Se, which interacted with glutathione, leading to the generation of ROS and damage to liver tissue. In addition, an increase in biochemical indicators of LPO in the blood of experimental animals was observed, which indicates damage to the urinary tract [40].

In another study, when studying the toxicity of SeNPs at high (4 mg/kg/day), medium (0.5 mg/kg/day) and low (0.05 mg/kg/day) doses, it was found that high doses of SeNPs cause an increase in the relative weight of the liver, changes in biochemical parameters of plasma and urine creatinine, an increase in urine pH, etc. [41].

Thus, despite the presence of a number of significant advantages of SeNPs over other Se-containing forms, in each specific case, it is important to determine the “therapeutic window” to minimize the negative effects of excess SeNPs.

## 3. SeNPs as Inducers of Various Forms of Cell Death Using the Example of Liver Cancer Cells

### 3.1. SeNPs as an Inducer of Apoptosis in Liver Cancer Cells

It is currently believed that apoptosis is one of the most important mechanisms of chemoprevention and chemotherapy with Se compounds, including SeNPs, which cause apoptotic death of liver cancer cells, which has been demonstrated in a number of studies [27,42,43,44,45,46,47,48,49,50].

It has been shown that ultrasmall (about 5 nm) SeNPs nanolyzed with polyethylene glycol promote mitochondrial depolarization, which leads to apoptotic HepG2 cell death [43].

In a comparative analysis of nanoparticles doped with and without sorafenib, approximately 100 nm in size using the example of HepG2 cells, it was found that the most effective was the nanocomplex of Se and sorafenib, which, at a concentration of 0.5 μg/mL contributed to the induction of early stages of apoptosis after 24 h, whereas increasing the concentration of the nanocomplex by five times led to the induction of late stages of apoptosis. SeNPs of both types promoted an increase in the expression of a number of pro-apoptotic genes, including *GADD34*, *BAK*, *BAX*, *PUMA*, *CASP-3* and *CASP-4* [45].

It was also demonstrated that siRNA with polyethylenimine (PEI)-modified SeNPs enhanced apoptosis of HepG2 cells by reducing the expression of heat-shock protein HSP70 mRNA, which was accompanied by the inhibition of the protective function of these cells and subsequent apoptotic death. DNA fragmentation and nuclear condensation were observed, which are important biochemical signs of cellular apoptosis. Treatment of HepG2 cells with various concentrations of this nanocomplex significantly increased the activity of caspase-3 and the cleavage of DNA repair enzyme PARP in a dose-dependent manner [48].

Laminarin polysaccharide-decorated SeNPs (LP-SeNPs) with a diameter of 60 nm also exhibited cytotoxicity towards HepG2 cells. Thus, after their treatment with various concentrations of LP-SeNPs (10, 20 and 40 μM) for 24 h, average increases in the total rate of apoptosis of 17.4, 20.9 and 30.9%, respectively, were observed. These processes were accompanied by increases in BAX mRNA expression and caspase-9 cleavage and a decrease in BCL-2 levels. The obtained data most likely indicate the activation of mitochondrially mediated apoptosis by SeNPs [36].

SeNPs synthesized using hawthorn fruit extract (HE) as a reducing and stabilizing agent (HE-SeNPs) at concentrations of 5, 10 and 20 μg/mL caused early and late stages of apoptotic death of HepG2 cells [49]. In this case, the rate of apoptosis increased to 7.3, 9.7 and 19.2%, respectively; there was an increase in intracellular levels of ROS, a decrease in membrane potential, an increase in the level of caspase-9 and a decrease in the level of mRNA of anti-apoptotic protein BCL-2. Thus, He-SeNPs induced intracellular oxidative stress and mitochondrial dysfunction by initiating apoptosis of HepG2 cells through the mitochondrial pathway.

It was found that BFP (triple-helix β-glucan)-SeNPs were more effective than naked SeNPs, significantly reducing proliferation and disrupting the cell cycle and apoptosis of HepG2 cells [50]. Here, BFP not only acted as a stabilizer of SeNPs but also activated death receptor-mediated and mitochondrially mediated apoptosis pathways in liver cancer cells. However, BFP itself exhibited negligible cytotoxic activity against HepG2 cells, even when cells were treated with a dose of 200 μg/mL for 72 h. Naked SeNPs had a slightly better effect. But the BFP-SeNP nanocomplex showed significant synergistic effects, including the inhibition of cell proliferation through cell cycle arrest in the S phase, condensation of nuclear chromatin and severe nuclear shrinkage, an increase in ROS, a decrease in mitochondrial membrane potential and activation of the endogenous mitochondrial apoptosis pathway [50].

### 3.2. SeNPs as Inducers of Autophagy in Liver Cancer Cells

Recently, more and more evidence has emerged indicating the important role of SeNPs in the treatment of cancer and various infectious diseases through the regulation of autophagy, which is one of the most important intracellular processes of the degradation system [51].

Using the example of the above-mentioned laminarin-coated SeNPs (LP-SeNPs), it was demonstrated that exposure of HepG2 cells to this nanocomplex for 12 h led to the activation of light chain 3-II (LC3-II) and p62, important participants in the processes occurring during autophagy [52]. LC3 is a soluble protein weighing 17 kDa associated with microtubules, the cytosolic form of which (LC3-I) is conjugated with phosphatidylethanolamine to form LC3-II, which is recruited to autophagosomal membranes, that is, autophagy begins with the formation of a phagophore closely associated with LC3-II [52]. The first autophagy adapter discovered in mammals is p62 [53,54]. Therefore, biochemical methods based on the analysis of LC3-II and p62 are key in assessing the degree of autophagy progression in mammalian cells. These data suggest that LP-SeNPs induce the activation of early autophagy but block the late phase of autophagy. Since the damaged organelles could not be cleared, there was a decrease in the fusion of autophagosomes with lysosomes and a decrease in the enzymatic activity of lysosomes, which ultimately worsened the apoptosis of HepG2 cells.

To date, sufficient data have not yet been accumulated to convincingly prove the role of SeNPs in the regulation of autophagy processes in liver cancer cells; however, it has been shown that after 24 h and especially after 48 h, SeNPs doped with sorafenib (SeSo) are able to reduce the expression of mTOR, AKT and PI3K [45]. It is known that mammalian rapamycin kinase mTOR is a negative central regulator of the autophagic process; therefore, inhibition of the PI3K/AKT/mTOR pathway leads to the activation of autophagy, which is also important evidence of the death of HepG2 cells through autophagy after 24 and 48 h of treatment with SeNPs and SeSo nanoparticles.

## 4. Signaling Pathways Activated by SeNPs in HCC

The signaling mechanisms that SeNPs can trigger in liver cancer cells have not been sufficiently studied. However, there are already some general trends to consider with respect to some of the signaling pathways in which SeNPs are involved. Among them, it is worth highlighting the phosphatidylinositol 3-kinase/protein kinase B/mammalian target of rapamycin (PI3K/AKT/mTOR), Wnt/β-catenin and RAS/RAF/UPR (unfolded protein response) signaling pathways during endoplasmic reticulum stress.

### 4.1. Participation of SeNPs in the Regulation of ER Stress in HCC

Endoplasmic reticulum stress (ER stress) is a typical molecular pathophysiological process that underlies many human diseases, for the development of which disruption of protein folding is of great importance, which can lead to the accumulation of inactive or chemically aggressive proteins in the lumen of the ER [55].

When assessing the cytotoxic effect of “naked” SeNPs, SeNPs coated with sorafenib (SeSo), with the drug sorafenib (So) using the example of HepG2 cells, it was found that all agents are able to activate UPR signaling pathways when exacerbating ER stress [45]. In this study, it was found that after 24 h of treatment of HepG2 cells with So and SeSo, there was an increase in the expression of the mRNA of transcription factor ATF-4, which is a key marker of the PERK (protein kinase RNA-like endoplasmic reticulum kinase) signaling pathway of the UPR [56,57,58,59,60]. However, no increase in the expression of this transcription factor was observed after treatment of cells with SeNPs. On the contrary, after treatment of HepG2 cells with SeNPs and SeSo, a noticeable decrease in the spliced form of transcription factor XBP1 (XBP1s), which can be considered a key marker of the IRE1α signaling pathway of the UPR, was observed [61,62,63].

Since this work established that treatment of HepG2 cells with SeNPs, So and SeSo for 24 h promoted apoptotic death, this most likely occurred, among other things, as a result of prolonged ER stress, which was accompanied by the activation of not the adaptive but pro-apoptotic UPR signaling pathways. The work also shows the connection of SeNPs and SeSo nanoparticles with the dose-dependent generation of various calcium signals (single pulses and calcium oscillations). At the same time, an increase in the expression of caspase-4 was observed, which is activated under ER stress conditions, including through the Ca^2+^ signaling pathway [64,65,66]. It is known that during ER stress, conformational changes and/or oligomerization of pro-apoptotic proteins BAX and BAK occur, an increase in expression of which was also recorded in this work [45], which can lead to damage to calcium stores in the ER and the release of calcium into the cytosol [67,68]. This, in turn, activates cysteine protease m-calpain, which further cleaves procaspase-12 to caspase-12, leading to the activation of apoptosis [64,65,66]. Caspase-12 has been identified in rodents, and in humans, its role is presumably played by caspase-4 [69].

**Figure 1:**
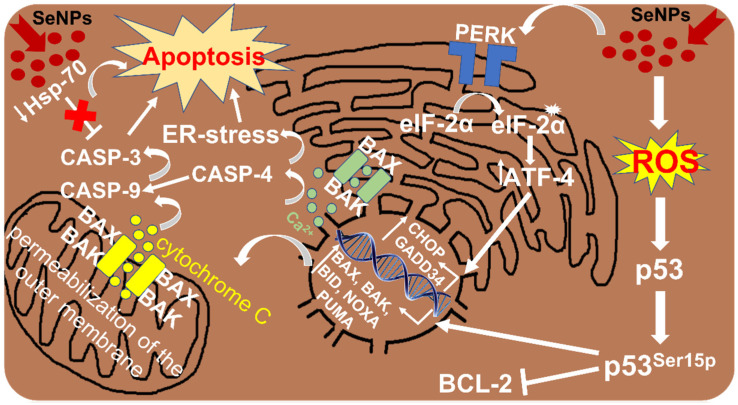
Activation of various signaling pathways leading to apoptosis of liver cancer cells after their treatment with SeNPs of various origins. Microsoft Paint was used to create the figure.

However, these experiments showed that SeNPs and SeSo are able to enhance the growth of pro-apoptotic markers *CHOP*, *BIM*, *PUMA*, *GADD34*, *BAX* and *BAK* mRNA expression and, at the same time, reduce the expression of anti-apoptotic gene BCL-2 [45]. It is well known that the activation of CHOP (CCAAT/enhancer-binding protein-homologous protein) occurs as a result of the activation of the UPR PERK signaling pathway described above [57,70]. Both anti-apoptotic gene *BCL-2* and pro-apoptotic *BIM* and *BAX* have been shown to be regulated by CHOP during ER stress, which suppresses BCL-2 expression, activates BIM and promotes BAX translocation into mitochondria [71,72]. These events may cause damage to the outer mitochondrial membrane, a decrease in membrane potential and the release of cytochrome C, which, together with the APAF-1 protein (apoptotic protease activating factor-1), is involved in the formation of the apoptosome. Next, CASP-9 is activated, which binds and activates pro-CASP-3 to form effector CASP-3, which leads to apoptosis of HepG2 cells. These processes are shown schematically in Figure 1.

SeNPs activate the PERK signaling pathway UPR as a cell response to increasing ER stress. In this case, the expression of the key marker of this signaling pathway, ATF-4, increases, which serves as a trigger for increased expression of genes such as *CHOP*, *GADD34*, *BAX* and *BAK*, which ultimately leads to the discovery of mitochondrial membrane permeability, the release of cytochrome C into the cytoplasm, activation of caspase- 9, apoptosome formation, caspase-3 activation and cell apoptosis. These processes are also accompanied by an increase in the concentration of cytosolic calcium, the activation of caspase 4 and further activation of caspases 9 and 3, which also leads to apoptosis of cancer cells. SeNPs can be a source of ROS of various natures, which leads to the activation (phosphorylation) of p53, which, in turn, blocks the expression of anti-apoptotic gene BCL-2 and activates increased expression of various pro-apoptotic genes of the BCL family (*BAX*, *BAK*, *PUMA*, *NOXA* and *BID*), which ultimately activates mitochondrially mediated apoptosis. SeNPs are able to enhance apoptosis of HepG2 cells by reducing the expression of heat-shock protein HSP70 mRNA, which is accompanied by DNA fragmentation, nuclear condensation, caspase-3 activation and apoptosis.

### 4.2. Participation of SeNPs in Wnt/β-Catenin Signaling in HCC

To date, there is sufficient evidence that Wnt/β-catenin signaling is active in HCC, since in liver tumors, HCC cells and macrophages are new sources of the Wnt ligand (from Wg (wingless) [73]. Mutations in various components of Wnt lead to hyperactivation of Wnt signaling in HCC [74,75], which is ultimately accompanied by the release of β-catenin [76,77]. The activated β-catenin then translocates into the nucleus, where it triggers the transcription of several target genes [75], e.g., the LGR5 (leucine-rich repeat-containing G) receptor is associated with HCC metastasis [78,79]. It is known that about 40–70% of HCCs have β-catenin in the nucleus, increasing the signaling activity of Wnt/β-catenin [17,80,81,82]; however, its nuclear accumulation is limited to late-stage HCC, whereas in earlier stages, it is mainly localized to the plasma membrane in complex with several cadherin family members. This study highlights the important function of β-catenin during HCC progression, as it promotes tumor cell survival by enhancing signaling of growth factor receptors such as EGFR [83].

**Figure 2:**
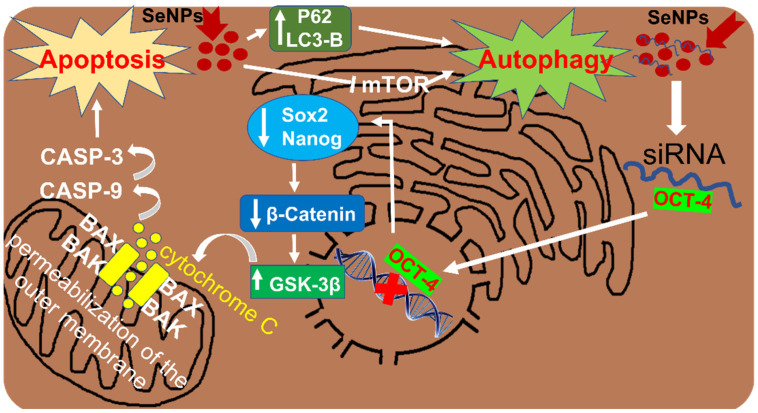
Activation of various signaling pathways leading to apoptosis and autophagy of liver cancer cells after their treatment with SeNPs of various origins. Microsoft Paint was used to create the figure.

It has been shown that peptide-modified SeNPs (RGDfC-SeNPs) used to deliver siRNA (anti-Oct4) to liver cells can activate Wnt/β-catenin signaling. RGD peptide binds to αvβ3 integrin, which is overexpressed in cancer cells [84]. It was found that knockdown of Oct4 resulted in significant suppression of the proteins Sox-2 (a transcription factor required to maintain self-renewal or pluripotency of undifferentiated embryonic stem cells) and Nanog (a transcription factor involved in self-renewal of undifferentiated embryonic stem cells). It is known that the regulation of Oct4 expression is associated with the activation of Wnt/β-catenin signaling [85]. Oct4 knockdown led to a decrease in the expression of β -catenin and GSK-3β, an important regulator of β-catenin phosphorylation. These processes are shown schematically in Figure 2.

SeNPs can be an inducer of Wnt/β-catenin signaling activation in liver cancer cells. As an siRNA carrier, they cause knockdown of important transcription factor Oct4, which is accompanied by a decrease in the expression of transcription factors Sox-2 and Nanog, as well as a further decrease in the expression of β-catenin and GSK-3β, an important regulator of β-catenin phosphorylation; the end result of all these events is mitochondrially mediated apoptosis of liver cancer cells. In addition, SeNPs are able to reduce the expression of mTOR, AKT and PI3K, as well as activate LC3-II and p62, important participants in the processes occurring during autophagy, which leads to the death of cancer cells through autophagy.

It was also shown that SeNPs in combination with or without quercetin had anti-inflammatory and hepatoprotective effects in a rat model of thioacetamide-induced hepatocellular carcinoma. Suppression of HCC progression in rats was due to increased oxidative stress and dysregulation of the oncogenic p53/β-catenin/cyclin D signaling pathway [39].

### 4.3. Involvement of SeNPs in the Regulation of PI3K/Akt/mTOR in HCC

The PI3K/AKT/mTOR signaling pathway is associated with autophagy and apoptosis and plays a vital role in both processes [86,87,88,89]. Some anticancer drugs induce apoptosis and autophagy by inhibiting the PI3K/Akt/mTOR pathway [90,91,92]. There is also evidence of an interaction between autophagy and apoptosis through the Bcl-2 family and the PI3K/Akt/mTOR signaling pathway [93]. To date, there is sufficient evidence that various SeNPs are able to inhibit the PI3K/Akt/mTOR signaling pathway in various cancer cells [45,94,95,96]. These processes are shown schematically in Figure 2.

Chemically synthesized SeNPs complexed with curcumin (Se@Cur) contributed to a significant enhancement of the Akt protein, inducing apoptosis in HepG2 cells and activating the PI3K/Akt/mTOR pathway [27]. At the same time, after 24 h and especially after 48 h, SeNPs doped with sorafenib (SeSo) are able to reduce the expression of mTOR, AKT and PI3K [45]. It is known that mammalian rapamycin kinase mTOR is a negative central regulator of the autophagic process; therefore, inhibition of the PI3K/AKT/mTOR pathway leads to the activation of autophagy, which is also important evidence of the death of HepG2 cells through autophagy after 24 and 48 h of treatment with SeNPs and SeSo nanoparticles.

### 4.4. SeNPs Regulate the Expression of ER-Resident Selenoproteins in HCC

Recently, enough work has been accumulated demonstrating the important role of various selenium-containing compounds in the regulation of selenoprotein expression in various cancer cells [25,97,98,99,100,101,102,103,104,105,106,107,108]. Thus, we previously studied the patterns of expression of seven ER-resident selenoproteins by SeNPs doped (SeSo) and undoped (SeNPs) with sorafenib in HepG2 cells [45]. After 24 h of treating cells with SeNPs, an almost twofold decrease in the mRNA of selenoprotein SELENOM was observed, and a trend towards a decrease in mRNA expression was recorded for two other selenoproteins, namely SELENOS and SELENOF. The SeSo nanocomplex promoted an almost twofold increase in the expression of SELENOT mRNA and significant increases in SELENON and DIO2.

To date, enough data have been accumulated to allow us to say with confidence that SeNPs actively regulate the expression of these oxidoreductases. Thus, in a study of the chemoprotective properties of SeNPs during hepatotoxicity in mice induced by cyclophosphamide, they were found to contribute to an increase in the levels of glutathione peroxidases, along with those of other enzymes [38]. Similar data were obtained in the treatment of HCC with SeNPs and SeNPs doped with quercetin [109]. Our results should probably be considered a protective antioxidant response, which turns out to be insufficient under conditions of prolonged ER stress and severe inhibition of key kinase pathways and ultimately leads to apoptotic cell death to varying degrees, with a predominance of apoptosis over necrosis in the case of incubation of cells with SeNPs and SeSo [45].

There is evidence that SeNPs can prevent HCC and protect liver cells from disturbances in glutathione homeostasis; they were found to reverse the decrease in the activity of glutathione peroxidases and glutathione S-transferases in the liver but had a subtle effect on the activity of glutathione reductases [13].

Chitosan-stabilized SeNPs (CS-SeNPs) also demonstrated acceptable antioxidant activity, increasing the concentration of glutathione peroxidases in the liver and the level of reduced glutathione in the serum of mice after injections with concavalin A, which indicates the hepatoprotective function of Cs-SeNPs, including protection against HCC [12].

## 5. Use of SeNPs to Improve the Effectiveness of Drugs in the Treatment of HCC

Radiation therapy and chemotherapy are both traditional methods of treating HCC. However, a number of problems are associated with multidrug resistance, targeted delivery and effective release of drugs, which makes these treatments highly ineffective.

Lipiodol chemoembolization (LCE) is widely used to treat patients with unresectable HCC [110], but it has poor control over sustained drug release [111,112]. In addition, So is a first-line systemic drug that significantly increases the survival of patients with HCC [113,114]. It inhibits tumor angiogenesis by blocking VEGF and PDGF receptors and tumor cell proliferation by blocking the B-Raf and c-Raf MAP kinase pathways to accelerate tumor cell apoptosis [115,116,117]. However, this drug has a number of serious side effects due to nonspecific absorption by normal tissues [118,119]. To improve anticancer effectiveness, an injectable nanosystem based on a thermosensitive hydrogel containing SeNPs and So was developed [120]. At the cellular level, it was shown that this hydrogel reduced the expression of CD34 and Ki67 and increased the growth of caspase-3 in tumor tissue. In vivo experiments showed that the developed thermosensitive nanosystem had great potential for local application, in addition to non-obvious side effects and low toxicity for nude mice. Treating mice with radiation therapy and the hydrogel together resulted in improvements in protein, serum creatinine, cholesterol and blood glucose levels, and no abnormalities in major organ function were observed in the mice.

In another study, in vivo experiments found that sorafenib-doped SeNPs restored the effects of TAA injections in animals; they reduced oxidative stress and caused the activation of pro-apoptotic genes encoding p53, BAX and caspase-3, as well as the inhibition of the expression of anti-apoptotic gene Bcl-2. Nanoselenium enhanced the effectiveness of sorafenib and reduced the resistance of liver cancer cells to drugs, affecting the mTOR and NF-kB pathways, in addition to contributing to a decrease in angiogenesis and metastasis [121].

Another common and effective chemotherapeutic drug, including for the treatment of HCC, is doxorubicin, but its clinical use is limited due to poor water solubility and off-target side effects [122,123]. To effectively deliver doxorubicin to liver malignancy, galactose-modified SeNPs loaded with doxorubicin were developed and demonstrated excellent cellular uptake into HepG2 cells via clathrin-mediated endocytosis, accompanied by rapid release of doxorubicin from the nanoparticles at pH 5.4. This nanocomplex more effectively suppressed HepG2 cell proliferation and induced apoptosis through activation of caspase-3 pathways. In vivo studies showed that the resulting nanocomplex reduced the percentage of Ki67-positive cancer cells. Since nuclear protein Ki67 is associated with cell proliferation, the nanocomplex reduced the proliferation of cancer cells and the size of tumors in mice. In addition, no pathological changes were observed in the heart, liver, spleen, lungs or kidneys of mice after treatment with the studied nanocomplex, which indicates the absence of its toxicity in vivo [124].

The main molecular mechanisms activated by various SeNPs in in vitro and in vivo HCC models are presented in Table 1.

## 6. Conclusions

This review presents currently available data on the molecular mechanisms regulating processes associated with the cytotoxic effects of SeNPs of various origins in hepatocellular carcinoma. The signaling cascades that are activated after exposure of liver cancer cells to selenium nanoparticles and the role of selenoproteins in these processes are described, the available biomarkers of HCC are described and the concept of intratumoral heterogeneity in HCC is considered. This review allows us to understand the selectivity of SeNPs against HCC, which is extremely important for the development of targeted hepatoprotective drugs based on them.

## Figures and Tables

**Table 1:** Molecular mechanisms activated by various SeNPs in in vitro and in vivo HCC models.

Nanoparticle Composition	Object of Study	Molecular Mechanisms Activated by Nanoparticles	Form of Cell Death	Ref.
SeNPs or SeSo Selenium nanoparticles or sorafenib + selenium nanoparticles	HepG2 cells	Promote an increase in the expression of a number of pro-apoptotic genes, including *GADD34*, *BAK*, *BAX*, *PUMA*, *CASP-3* and *CASP-4*; activate ER stress through the PERK signaling pathway; cause dose-dependent generation of various calcium signals	Apoptosis	[45]
Cur-SeNPs Curcumin + selenium nanoparticles	HepG2 cells	Activate the PI3K/Akt/mTOR pathway	Apoptosis	[27]
siRNA-PEI-SeNPs Small interfering RNA + polyethylenimine + selenium nanoparticles	HepG2 cells	Reduce the expression of HSP70; increase the activity of CASP-3 and the cleavage PARP	Apoptosis	[48]
LP-SeNPs Laminarin + selenium nanoparticles	HepG2 cells	Increase BAX mRNA expression and CASP-9 cleavage; decrease BCL-2 levels	Apoptosis	[49]
HE-SeNPs Hawthorn fruit extract + selenium nanoparticles	HepG2 cells	Increase the level of CASP-9; decrease the level of BCL-2; induce intracellular oxidative stress and mitochondrial dysfunction	Apoptosis	[50]
BFP-SeNPs Triple-helix β-glucan + selenium nanoparticles	HepG2 cells	Inhibit cell proliferation through cell cycle arrest in the S phase; cause condensation of nuclear chromatin and severe nuclear shrinkage; increase ROS; decrease mitochondrial membrane potential	Apoptosis	[51]
siRNA-RGDfC-SeNPs Small interfering RNA + peptide + selenium nanoparticles	HepG2 cells	KD of Oct4, which is accompanied by a decrease in the expression of Sox-2, Nanog, β-catenin and GSK-3β; activate Wnt/β-catenin signaling; reduce the expression of mTOR, AKT and PI3K; activate LC3-II and p62	Autophagy	[86]
QCT-SeNPs Quercetin + selenium nanoparticles	Rat model of TAA-induced HCC	Increase oxidative stress; dysregulate the oncogenic p53/β-catenin/cyclin D signaling pathway	Apoptosis or autophagy	[39]
So + SeNPs Sorafenib + selenium nanoparticles or sorafenib + selenium nanoparticles + radiation	HepG2 cells or mice with HCC	Reduce the expression of CD34 and Ki67; increase the growth of CASP-3. With radiation, hydrogel led to improved protein, se-rum creatinine, cholesterol and blood glucose levels.	Apoptosis	[120]
So + SeNPs Sorafenib + selenium nanoparticles	Mouse model of TAA-induced HCC	Decrease angiogenesis and metastasis, affecting the mTOR and NF-kB pathways	Apoptosis	[121]
DOX + HA-SeNPs Doxorubicin + hyaluronic acid + selenium nanoparticles	HepG2 cells	Suppress proliferation; promote the production of ROS; induce apoptosis through activation of CASP-3 pathways	Apoptosis	[124]

Akt—protein kinase B; BAK—Bcl-2 homologous antagonist/killer; BAX—Bcl-2-like protein 4; Bcl-2—B-cell lymphoma 2; CASP—caspase; CD34—transmembrane phosphoglycoprotein encoded by the CD34 gene; GADD34—growth arrest and DNA damage gene; GSK-3β—glycogen synthase kinase-3 beta; KD—knockdown; Ki67—antigen Kiel 67; LC3-II—autophagosomal marker; mTOR—mammalian target of rapamycin; NF-kB—nuclear factor kappa light-chain enhancer of activated B cells; Oct4—octamer-binding transcription factor 4; p53—tumor protein P53; PEI—polyethylenimine; PERK—protein kinase RNA-like endoplasmic reticulum kinase; PI3K—phosphoinositide 3-kinase; PUMA—P53 upregulated modulator of apoptosis; ROS—reactive oxygen species; TAA—thioacetamide; Wnt—created from the names wingless and Int-1.

## Data Availability

Not applicable.
